# Fabrication and characterization of antimicrobial wound dressing nanofibrous materials by PVA-betel leaf extract

**DOI:** 10.1016/j.heliyon.2023.e17961

**Published:** 2023-07-05

**Authors:** Md. Washim Akram, Mohammad Mohsin Ul Hoque, Md. Sumon Miah, Md. Abdus Shahid, Md. Firoz Hossain, Sayed Hasan Mahmud

**Affiliations:** a Department of Textile Engineering, National Institute of Textile Engineering & Research (NITER), Nayarhat, Savar, Dhaka, Bangladesh; b Department of Textile Engineering, Dhaka University of Engineering & Technology (DUET), Gazipur, Dhaka, Bangladesh

**Keywords:** Antibacterial assay, Betel leaf, Electrospinning, Nanomat, Wound healing

## Abstract

This present study involves the formation and investigation of the characteristics of a fabricated mat from a PVA-betel leaf mixture. Under ideal processing parameters, nanofibrous mat is synthesized from the PVA-betel leaf blended solution by using the electrospinning technique. Afterwards, the produced nanofibrous mat is assessed for its thermal, antibacterial, morphological, moisture management and chemical interaction behavior using thermogravimetric analysis (TGA), antibacterial assay, scanning electron microscope (SEM), moisture management tester (MMT) and Fourier-transform infrared spectroscopy (FTIR) respectively. The antibacterial action against *Staphylococcus aureus* and *Escherichia coli* bacteria has been assessed using the agar diffusion technique, which reveals the creation of zones of inhibition with a value of about 20 mm. Besides, the fabricated nanomat reveals an average diameter of 183.4 nm with improved moisture and thermal characteristics. Furthermore, the generated nanofibrous mat has all the necessary components, as evidenced by the distinctive peaks in the FTIR spectra. Hence, the recently developed nanofibrous mat exhibits promising potential as a suitable material for wound dressing applications.

## 1 Introduction

Recently, the medical sector has expressed greater interest in wound dressing products that contain herbal medicines rather than those that use metal-based nanomaterials to achieve the anti-fungal and antibacterial properties. Even in the present day, the use of herbal medicines for basic healthcare continues to include the wound healing and burn injuries as well as antifungal, antiviral and antibacterial applications for infections of the skin [1,2]. The provision of a micro-moist climate, shielding the wounds from bacterial invasion, limiting tissue damage to the wound surface, preventing wound dehydration and being biocompatible and biodegradable are some important requirements for a material to be used as a wound dressing material. The replacement of metallic particles in the existing dressing products due to the inherent therapeutic properties of natural herbs' makes the creation of such materials of tremendous relevance. Aromatic plants have historically been employed in traditional medicine and to increase food life span due to their ability to suppress the growth of bacteria, fungus and yeasts [3]. The researchers studying contagious illnesses have been very interested in substances with biological activity from natural sources [4].

Electrospun nanofiber materials have a diverse range of applications in the biomedical field, including wound dressing, tissue engineering and drug development. These prominent characteristics like biocompatibility, high porous structure as well as excellent pore interconnectivity and surface area are potentially liable [[5], [6], [7], [8]]. This technique is highly versatile, affordable, capable of producing continuous nanofibers, easy to functionalize the surface, industrially applicable and unique in that it can produce fibers ranging in size from several micrometers to nanometers when electrical forces are applied [[8], [9], [10]]. The porous nature of nanofibrous mats and their high active contact surface provided by their huge surface to volume ratio encourage cell adhesion, proliferation and transformation [7,[11], [12], [13], [14]]. Additionally, it achieves consistent adhesion on a moist wound surface without any fluid buildup, which enables it to satisfy demands for greater gas permeability and wound protection from dehydration and infection [15]. To provide nanofibers with antibacterial properties, metal nanoparticles are frequently used [16,17]. Natural antimicrobes are now used in their place because they are expected to be environmentally friendly and less likely to grow resistant to bacteria. This is because synthetic antibiotics and these nanoparticles have detrimental effects on the environment, as well as human health issues. Due to the intrinsic therapeutic characteristics of natural plant extract and its fabrication through the electrospinning technique, there is potential for usage in different biomedical applications, particularly wound dressings [15,[18], [19], [20], [21]].

Customer awareness of the hygienic and possibly hazardous effects of microbes has increased recently, which is why the need for antimicrobial treatments is rising steadily [22]. Although synthetic antimicrobial treatments like titanium dioxide and zinc oxide are efficient against a wide variety of bacteria, they also have certain negative side effects such as increased skin sensitivity and pollution [23]. As an outcome, adding antibacterial qualities to materials for wound dressings derived from natural sources [23] is seen as a key strategy for satisfying consumer demand [24]. Organic antibacterial agents can be found in a number of secondary metabolites that have been identified in plants. Each chemical has a unique antibacterial action that prevents germs from growing. In the protoplasm, the antibacterial action performs due to the toxin behavior of phenol that enables rupturing the cell wall and enhances the initiation process of controlling the mechanism of protein mass, selective permeability which causes disrupted bacterial cells [25,26]. By preventing the synthesis of nucleic acids, altering the function of cytoplasmic membranes, obstructing energy metabolism, preventing the formation of biofilms, preventing the permeability of cell walls, causing lyse bacterial cells as well as flavonoids work to prevent the growth of bacteria [[27], [28], [29], [30], [31]]. On the contrary, Alkaloids prevent bacterial development and permeability to membranes, restrain the production of nucleic acids and affecting the energy metabolic system of microbe's wall, these causes induce cell lysis. The bio-synthesis mechanism of bacterial cell walls also interrupted by alkaloids [[32], [33], [34]]. Moreover, Tannins can coagulate bacterial protoplasm, which causes proteins to precipitate and bind to one another, inhibiting the development of bacterial cell walls [[35], [36], [37]]. The ability to get such qualities may be obtained from a variety of natural sources, including collagen, chitosan, neem and tulsi. One of the many useful medicinal plants is *Piper betel*, whose leaves have been utilized for a variety of therapeutic reasons. The plant family *Piperaceae*, which includes *Piper betel*, is also known as Paan in India and sub-continent countries. Since ancient times, chewing has involved wrapping fresh betel leaf leaves with areca nut, mineral slaked lime, catechu, flavoring agents and spices [38]. As a popular after-meal mouth refresher, betel leaf (*Piperaceae*) leaves are abundantly produced in Bangladesh and other Southeast Asian nations. Betel is an evergreen dioecius plant that is widely grown across India. It requires warm, wet conditions to flourish [39].

D. Chakraborty et al. tested the antibacterial and antioxidant capacities of various *Piper betel* leaf extracts against four different pathogens, including *Pseudomonas aeruginosa Streptococcus pyogens*, *Staphylococcus aureus (S. aureus)* and *Escherichia coli (E. coli)*. The spherical gram positive bacteria *Streptococcus pyogensis* produced favorable results [40]. The leaves also include *hydroxychavicol*, *chavicol*, *chavibetol*, *eugenol*, *piperol A*, *methylpiperbetol* and *piperol*, among other physiologically active compounds. *Betel* oil, a volatile oil, is an essential component of the leaf [41]. Anam Razzaq et al. synthesized cephradine-loaded gelatin/polyvinyl alcohol electrospun nanofibers for diabetic wound treatment. The effectiveness of the drug-loaded nanofibers was assessed both *in vitro* and *in vivo* in the study. The findings showed that when compared to other treatments, the drug-loaded nanofibers exhibited much stronger antibacterial activity. Additionally, by successfully removing bacterial infections, the use of these nanofibers accelerated the healing of chronic wounds. Overall, the results point to the drug-loaded nanofibers as a safer and more efficient method for treating diabetic wounds [42]. The production and characterization of electrospun nanofibers produced from chitosan (CS) and polyvinyl alcohol (PVA) and loaded with cefadroxil monohydrate (CFX), a broad-spectrum antibiotic, were the topic of a study by Haroon Iqbal et al. [43]. The goal of the study was to determine if these electrospun CFX-loaded nanofibers might be employed as a transdermal drug delivery system to speed up the healing of wounds and successfully treat skin infections brought on by *S. aureus*, including resistant strains. The results indicated that the electrospun CFX-loaded CPNFs would represent a viable and economical method for promoting wound healing and treating skin infections brought on by *S. aureus*. However, in our study, we have utilized a green material (betel leaf) to develop a sustainable wound dressing employed with natural compounds like phenols, flavonoids, alkaloids, tannins, saponins, terpenoids, glycosides and steroids [44,45].

To treat cytotoxic diabetic rats, a chitosan-PVA mixed nano-fiber mat was synthesized as a dressing material by the researcher Majd et al. as well as noticed that the wound healing of animals was significantly accelerated by the fiber mat [46]. J. Adhikari used electrospinning to create a tissue engineering mat out of honey and betel with chitosan and polycaprolactone [47]. A. Ali et al. recently generated an electrospun nanomat with silver incorporated in it utilizing an antibacterial bi-layered polyvinyl alcohol (PVA)-chitosan mix wound dressing material incorporating *Azadirachta indica* (neem) extract [48] as well as nigella extraction was used to make an electrospun nanomat embedded with silver [49] by A. Ali et al. Shahid et al. electrospun a mixture of PVA, turmeric, honey and curcumin longa extract to create a wound healing products [50].

In this research, it was recommended that, under ideal processing circumstances, a single-layered approach was used to create a nanofibrous mat made of a PVA-Betel leaf mixture. Investigations have been conducted on the produced sample's shape, bonding behavior, antimicrobial action against *S. aureus* and *E. coli* bacteria and moisture management capabilities. The developed nanofibrous mat holds promise as a suitable material for wound dressing in clinical applications.

## 2 Materials & methodology

### 2.1 Materials

Locally collected fresh Betel leaves, 99% hydrolyzed polyvinyl alcohol, molecular weight (MW) 115,000 DP were used as carrier and 99% pure Ethanol (C_2_H_5_OH) with MW of 46.07 g/mol was sourced from Merck, Germany and distilled water was used as solvent. All of the chemicals were analytically pure and did not require any further purification.

### 2.2 Betel leaf extraction

Initially, we collected fresh betel leaves and rinsed them with normal water. Then the betel leaves were cut into small pieces and dried in the sunlight until the moisture of those peels have been fully evaporated (for at least 7 days). Then the dried betel leaves were grinded so that the betel leaves finely turned into fine powder and alcoholic (ethanol) extraction was followed at a ratio of 1:10 to extract the bioactive constituents of betel leaves maintaining the duration of 48 h at room temperature.

### 2.3 Fabrication of PVA/Betel leaf nanomat

**Fig. 1:**
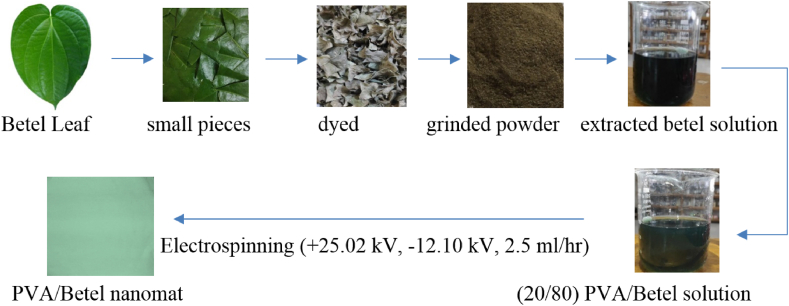
Diagram illustrating the development of PVA/Betel leaf nanomats.

PVA solution was prepared at a 10% (w/v) concentration at 80 °C using water as solvent. Besides, electrospinning solution was prepared following the PVA and leaf extract ratio of 20:80. Prepared solution was then loaded into a syringe of 30 mL and placed carefully to the solution pump. Later, electrospinning parameters in terms of applied voltage (+25.02 KV, −12.10 KV), solution rate (2.5 mL/h), heater power (0.38 kW) and collector distance (15 cm) were set to develop desired nanofiber (Fig. 1).

### 2.4 Statistical analysis

For statistical analyses and related graphs, including thermal analysis, FTIR and average cross sectional diameter of developed sample, we used Origin Pro 8.5.

### 2.5 Characterizations

#### 2.5.1 Thermal properties

We have used the ASTM E1131 test standard for TGA. A thermal analyzer (SDT 650, Discovery, USA) was used to evaluate the developed mats' thermal behavior or thermogravimetric analysis (TGA), at temperatures between 50 °C and 375 °C with 50 °C/min constant heating. A sample of 7.5 mg was obtained, heated to determine its properties and then analyzed.

#### 2.5.2 Moisture management

According to AATCC 195–2009, a moisture management tester (MMT) (Model-M290, SDL Atlas, Origin-UK) was used to assess the created mat's moisture management capabilities. A number of measures were made in order to categorize the mat based on how it interacted with fluids, including the mat's spreading speed, wetting time, maximum wetted area, absorption time, cumulative one-way transport capacity (R) and overall moisture management capacity.

#### 2.5.3 Antibacterial assay

The effectiveness of the developed samples' antibacterial activities against both *S. aureus* & *E. coli* bacteria was evaluated using the disc diffusion technique (ASTM E2149-20). Besides, TSA plates using 0.5 × 106 colony forming units (CFUs)/mL bacterial culture growth for a qualitative disk diffusion test. The 13 mm diameter nanofibrous mat was pelletized on an agar plate to provide the sample for the research, which was then left in the incubator at 37 °C overnight to assessed the zone of inhibition of developed sample.

#### 2.5.4 SEM analysis

A SU 1510, Hitachi, Japan was used to investigate the morphological arrangement of nanofibers in the samples for the magnification of 0.5–3.5 K times under a voltage of 5 kV.

#### 2.5.5 FTIR

We have used the ASTM E168-16 test standard to investigate the chemical behaviors of developed mat through a Fourier transforms infrared spectroscopy (IRPrestige21, Shimadzu Corporation, Japan). The spectra of the fabricated samples were captured with a resolution of 4 cm^−1^ and a range of 500–4000 cm^−1^.

## 3 Result and discussions

### 3.1 TGA analysis

**Fig. 2:**
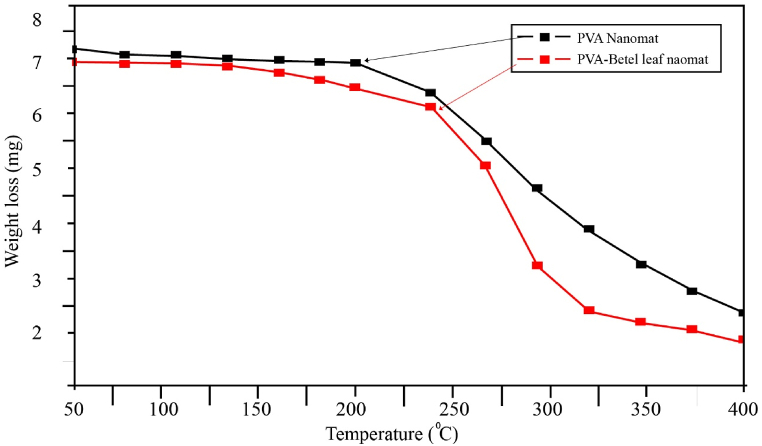
TGA of PVA nanomat and PVA-betel leaf nanomat.

Thermogravimetric analysis is used to analyze the weight changes that occur when a material is exposed to steady heat in order to measure its thermal stability. Fig. 2 displays the temperature behavior of implanted PVA nanomat and PVA-betel leaf nanomat. The addition of betel leaf extracts causes improved early degradation at a higher temperature of 240 °C with a slight weight loss, even though PVA nanofibrous possess melting temperature 190 °C or 227 °C because of having greater amorphous region [50]. The interaction between betel leaf and PVA polymer chains, resulting the segmental movement becomes attenuated and demanded high energy for the movement of polymer chains, prompted the increased heat stability of PVA-betel leaf. As a result, PVA-betel leaf nanomat has significantly higher thermal stability than PVA nanofiber.

### 3.2 Moisture management test result

**Fig. 3:**
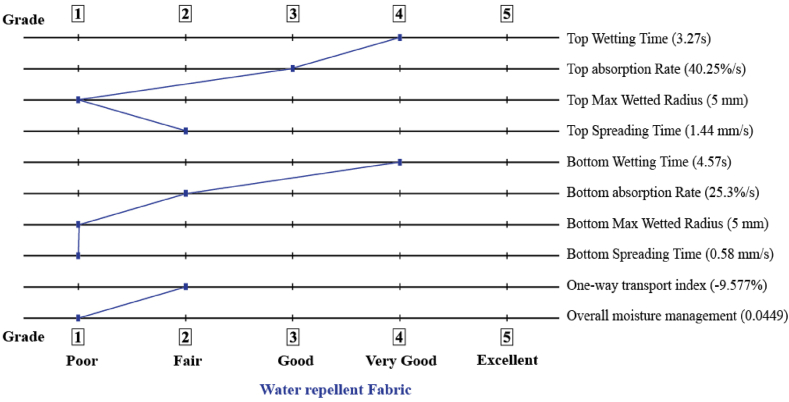
Moisture management characteristics (PVA-betel leaf nanomat).

**Fig. 4:**
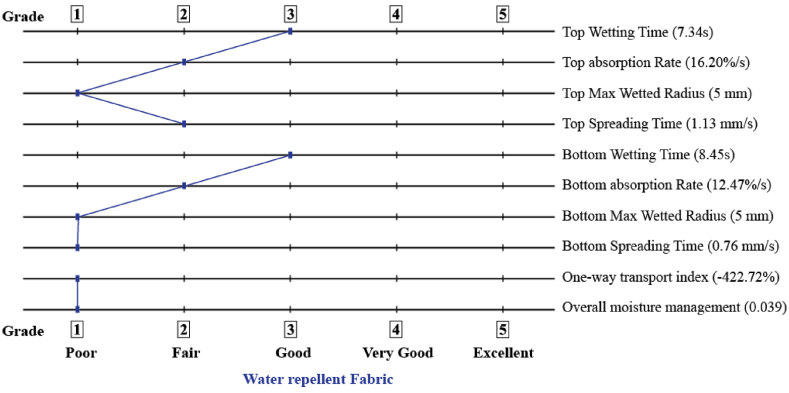
Moisture management characteristics (PVA nanomat).

**Fig. 5:**
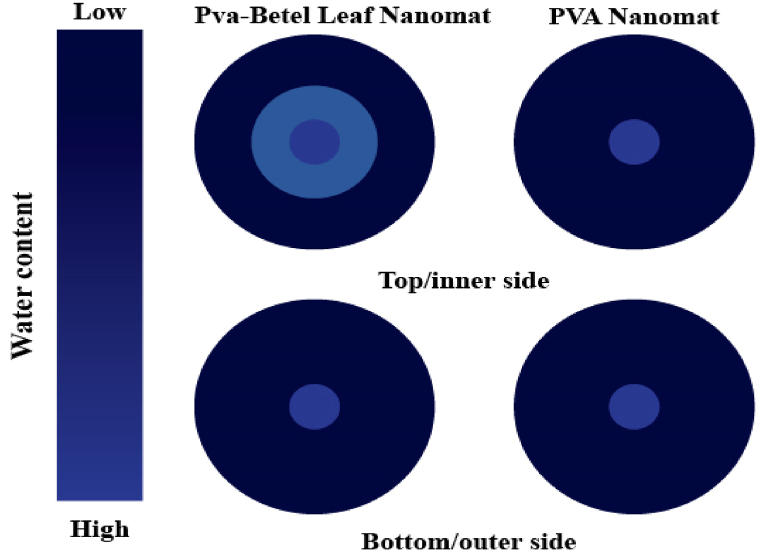
Water location vs time diagram of the fabricated sample (test time: 2 min).

Dressing materials are tested for their ability to control moisture in order to demonstrate how quickly liquids are transferred from the skin to the environment. Fig. 3, Fig. 4, reveal that PVA nanomat has a longer wetting time than PVA-betel leaf nanomat, which might be a consequence of PVA's sticky attributes and its lower absorption rate (16.20%). On the other hand, PVA-betel leaf nanomat wetting time is graded 4, indicating its fast-wetting nature in accordance with AATCC standards. When betel leaf extracts are added to the PVA polymer in the developed mat, the absorption rate is enhanced due to the absorptive quality of the betel leaf, which promotes the wound area to dry more quickly. Furthermore, as shown in Fig. 5, the maximum wetted area is 5 mm for both surfaces of the PVA-betel leaf nanomat, but the outer surface (PVA layer) of the PVA-betel leaf nanomat exhibits a lower absorption rate because of the adhesive nature of the PVA polymer, which limits moisture adherence through it. On the contrary, the inner surface (betel leaf layer) nanomat absorbs more than the outer surface (PVA layer), which is of good quality (40.25%), allowing for one-way transport of liquids from the wound area and promoting rapid healing. However, the values of overall moisture management capacity (OMMC) and one-way transfer capacity (OMTC) have been determined utilizing all indicators indicating the mat is water-resistant.

### 3.3 Antibacterial test result

**Fig. 6:**
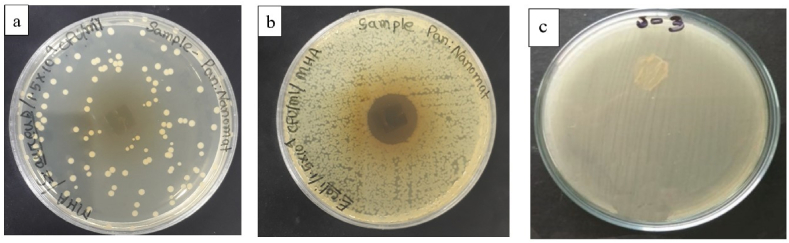
(A), (b) Antibacterial action of PVA-betel leaf nanomat, (c) PVA nanomat.

**Fig. 7:**
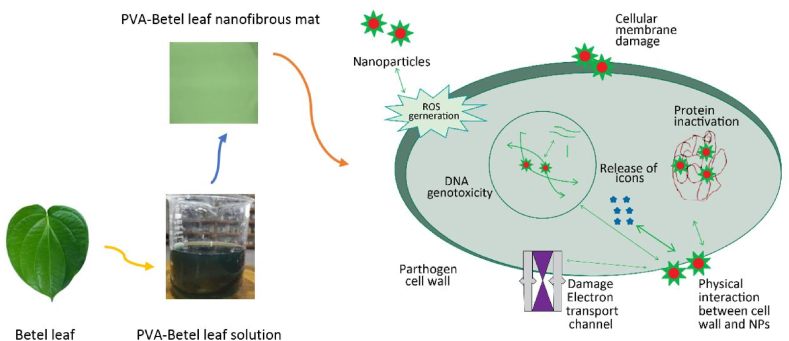
Mechanism of cell membrane damaging action of nanofibrous mat in gram-positive bacterial cell [56,57].

**Fig. 8:**
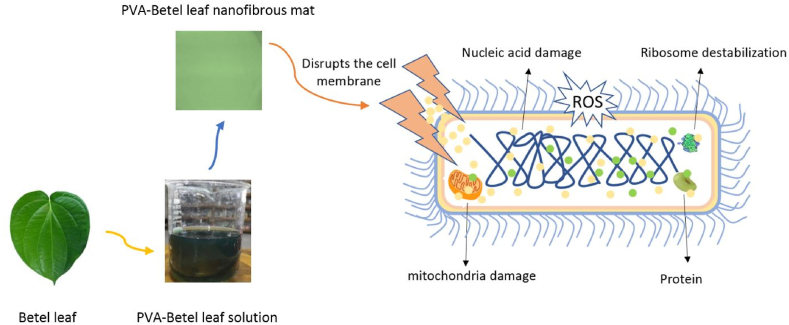
Mechanism of cell membrane damaging action of nanofibrous mat in gram-negative bacterial cell [58].

The disc diffusion technique was used to examine a PVA-betel leaf nanofibrous mat's antibacterial effectiveness. At a bacterial concentration of 1.5 × 10^4^ CFU/mL, the test assessed how well the mat protected against gram-positive bacteria (*S. aureus*) and gram-negative bacteria (*E. coli*). Fig. 6(a–c) shows how a PVA-betel leaf nanofibrous mat and a PVA nanomat produce inhibitory zones and exhibit antibacterial activity. In comparison to PVA nanomat, which did not generate a zone of inhibition against *S. aureus* or *E. coli*, PVA-betel leaf nanofibrous mat demonstrated an exceptional 20 mm zone of inhibition against these bacteria that is clearly visible in Fig. 6(a–c). This potential activity is a result of the presence of various active compounds (phenols, flavonoids, alkaloids, tannins, saponins, terpenoids, glycosides and steroids) present in betel leaves. Protoplasmic phenol compounds damage the bacterial cell wall, acting as a toxin. They get through the wall and interfere with vital processes such protein composition regulation, active transport and selective permeability. The result is bacterial cell lysis and distortion. Additionally, the antibacterial effects of Piper betel leaf extract are enhanced by flavonoids and tannins. Flavonoids interfere with the potassium concentration of gram-positive bacteria, which disrupts the organism's cytoplasm membrane. In Fig. 7, Fig. 8 depicts the antibacterial mechanism of the PVA-betel leaf nanofibrous mat where the developed mat disrupts and penetrate the cell membrane of the bacteria, which causes significant negative impacts on cell metabolic activities, like proteins inactivation, damaging DNA, restricts nucleic acid synthesis, damage electron transform channel and metabolic pathway which leads to the death of bacteria [44,45,[51], [52], [53], [54]]. Furthermore, the meshed structures of the material exist at nanoscales, which can efficiently eliminate nearby diseases by preventing bacteria from entering external environments [55].

### 3.4 SEM analysis

**Fig. 9:**
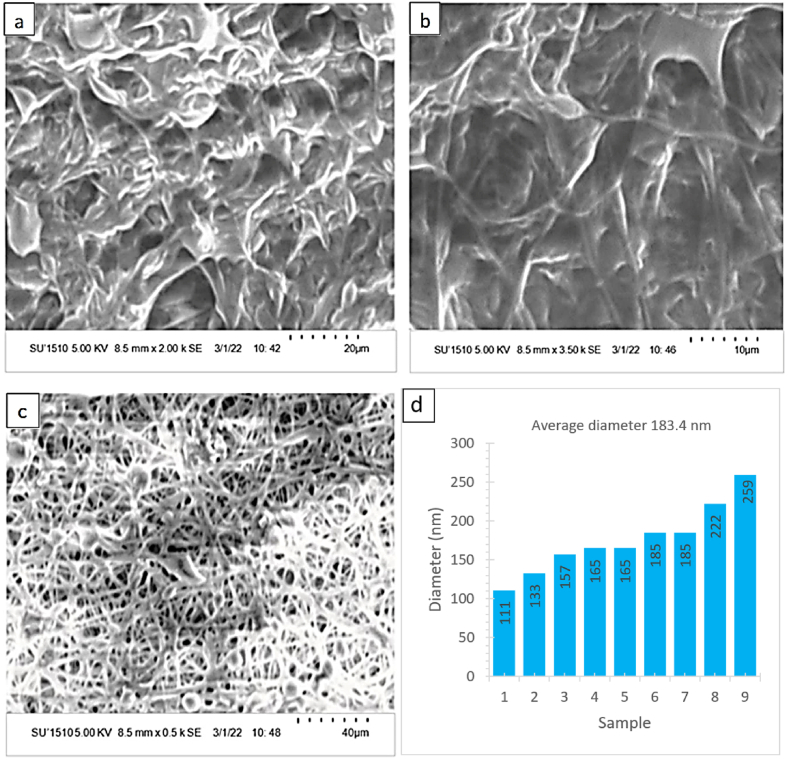
(a, b, c) Surface morphology and (d) average cross sectional diameter of the PVA-betel leaf nanomat.

In Fig. 9 (a, b, c) the morphological structure along with the porous nature is exhibits by the SEM images of developed mat. This porous nature may result from the nanofibers' mesh structure, which are created for random overlapping nature of fibrous on it. These tiny porous improve the efficacy of wound dressing materials by enabling oxygen permeability & proper diffusion of air to the skin. Moreover, it permits the air ventilation with proper restriction of germs penetration from the wound area [59,60].

In this study, ten samples were evaluated in different portion and in Fig. 9 (d) the fiber diameters were determined at 111 and 259 nm (minimum and maximum respectively) whereas the average diameter of 183.4 nm.

### 3.5 FTIR analysis

**Fig. 10:**
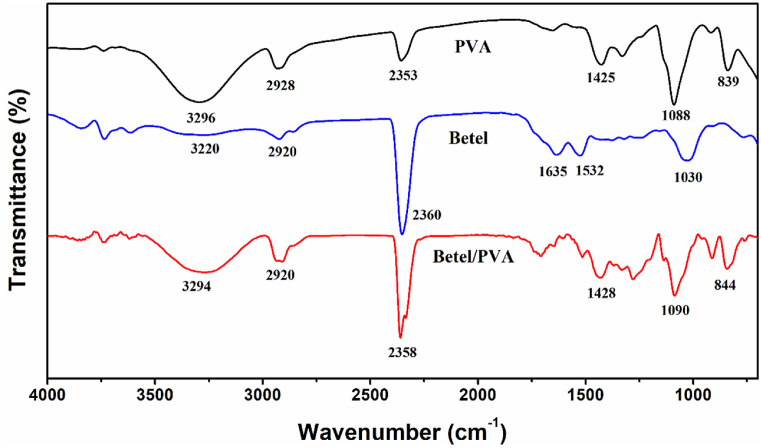
FTIR band spectra of PVA and PVA-betel leaf nanomat.

Fourier transfer infrared spectroscopy (FTIR) has revealed the functional groups of PVA, betel leaf & PVA/Betel leaf nanofibrous developed samples, as shown in Fig. 10. The functional group is identified between the range of 4000–1450 cm^−1^ and the region of 1450–500 cm^−1^, which corresponds to the finger print region. Whereas, the characteristic peaks of PVA polymer at 3294, 2920 and 1090 cm^−1^ correspond to O–H stretching, C–H stretching and C-O-C stretching respectively [61].

The major characteristic peaks for betel leaf extract are at 1030, 1532, 1635, 2360, 2920 and 3220 cm^−1^ as shown in Fig. 10. The peak at 1635 cm^−1^ is characteristic of C=O stretching vibrations in ketones, aldehydes and carboxylic acids or may be due to the amine group deformation, most likely from indole-3-acetic acid. Other significant transmittance peaks at 1025, 1532, 2360, 2920 and 3220 cm^−1^ correspond to *C*–*O*–C stretching, *N*–H stretching (presence of primary amine), *C*–H stretching and the presence of –OH/N–H bond stretching, respectively [62,63]. In the case of the composite film (PVA-Betel), all major absorption peaks of PVA (3294, 2920 and 1090 cm^−1^) and betel leaf extract (2360 cm^−1^) are observed with a slight shift or peak broadening. The peak shifting from 2360 cm^−1^ to 2358 cm^−1^ is corresponds to hydrogen bond formation between amine groups (betel leaf) and –OH groups (PVA) in composite film.

## 4 Conclusion

In this study, PVA-betel leaf nanofibrous mat has been developed by following relevant parameter as well as have been controlled faithfully during the fabrication process. Thermal behavior as well as moisture absorption, antibacterial activity & morphological characteristics have been tested under proper testing conditions. It is found that the antibacterial activity of a developed nanofibrous mat exhibits a significant ZOI value (20 mm) against both against *S. aureus* or *E. coli* bacteria and will provide outstanding protection during the wound healing process whereas there is no zone of inhibition is formed in case of PVA nanomat. The moisture management performance of the PVA-betel leaf nanomat is satisfactory level that allows the wetted wound liquid to easily pass from the wound area to the wound dressing materials and thus allows quick drying of the wet wound. Furthermore, the tiny porous structure of the developed mat enhances the air ventilation but restricts the dust and bacteria penetration from the external environment to the skin through the tiny pores present on it resulting enhance the efficacy of the wound dressing materials. The overall findings figure out the potential bacterial resistance and improved moisture management capabilities of the produced nanomat. As a result, PVA-betel leaf based nanofibrous mat can be employed as a wound dressing and to a great extent as a biodegradable, bio-based and antibacterial material.

## 5 Limitations of the study

Future work may be focused on potential impact of this study in terms of therapeutic regimen that specifies the dosage, the schedule and the duration of treatment in living organism. In our study, the number of pathogens is two types, in future that should be optimized to test in a broad range and *in vivo* studies and pharmacokinetics in wound management can be assessed and the evaluation of cytotoxic results can be a further scope of study.

## Author contribution statement

Md. Washim Akram: Conceived and design the experiments; performed the experiments; wrote the paper.

Mohammad Mohsin Ul Hoque: Performed the experiments.

Md. Sumon Miah, Md. Abdus Shahid: Contributed reagents, materials, analysis tools or data.

Md. Firoz Hossain: Analyzed and interpreted the data.

Sayed Hasan Mahmud: Analyzed and interpreted the data; wrote the paper.

## Data availability statement

No data was used for the research described in the article.

## Declaration of competing interest

The authors declare that they have no known competing financial interests or personal relationships that could have appeared to influence the work reported in this paper.
